# Selective neck dissection for treating node-positive necks in cases of squamous cell carcinoma of the upper aerodigestive tract

**DOI:** 10.1590/S1516-31802008000200009

**Published:** 2008-03-06

**Authors:** Jamile Karina Antonio, Marcelo Benedito Menezes, Norberto Kodi Kavabata, Antonio Augusto Tupinambá Bertelli, William Kikuchi, Antonio José Gonçalves

**Keywords:** Neck dissection, Head and neck neoplasms, Carcinoma, squamous cell, Prognosis, Lymphatic metastasis, Esvaziamento cervical, Neoplasias de cabeça e pescoço, Carcinoma de células escamosas, Prognóstico, Metástase linfática

## Abstract

**CONTEXT AND OBJECTIVE:**

Modified radical neck dissection (MRND) is the classical treatment for neck metastases of squamous cell carcinoma (SCC) of the upper aerodigestive tract. However, it may still be accompanied by significant sequelae. One alternative for this treatment would be selective neck dissection (SND), which has a lower incidence of sequelae. The aim of this study was to define which neck metastasis cases would really be suitable candidates for SND.

**DESIGN AND SETTING:**

Retrospective clinical-surgical trial at the Division of Head and Neck Surgery, Faculdade de Ciências Médicas da Santa Casa de São Paulo (FCMSCSP).

**METHODS:**

We retrospectively studied 67 patients with SCC of the upper aerodigestive tract, divided into two groups: 1) 47 patients treated by means of SND (node-negative or node-positive), 2) 20 patients treated by means of MRND (all node-positive).

**RESULTS:**

Our results demonstrated that there was no difference between the patients treated with SND or MRND in relation to disease evolution, and that the main prognostic factor was lymph node involvement. We observed that patients with pharyngeal SCC and older patients presented worse evolution and would probably not be suitable candidates for SND.

**CONCLUSIONS:**

SND may be a good option for treating node-positive necks in selected cases.

## RESUMO

**CONTEXTO E OBJETIVO:**

O esvaziamento cervical radical modificado (ECRM) é o tratamento clássico para as metástases cervicais do carcinoma espinocelular (CEC) do trato aerodigestório alto (TADA). Este procedimento é considerado oncologicamente satisfatório, porém pode ser acompanhado de seqüelas significativas devido à extensão do procedimento e a grande manipulação de estruturas nobres, especialmente as nervosas. Assim, tem sido proposto o esvaziamento cervical seletivo (ECS) para o tratamento de casos selecionados, pN1, pN2 sem ruptura capsular, minimizando, ou mesmo evitando, deste modo, as seqüelas do ECRM. O grande questionamento atual é a definição de quais casos seriam eleitos para este procedimento, sem alterar o resultado oncológico de médio e longo prazo.

**TIPO DE ESTUDO E LOCAL:**

Estudo clínico retrospectivo realizado na Disciplina de Cirurgia de Cabeça e Pescoço do Departamento de Cirurgia da Faculdade de Ciências Médicas da Santa Casa de São Paulo.

**MÉTODOS:**

Estudamos 67 doentes portadores de CEC do TADA de 1990 a 2001 dividindo-os em dois grupos: 1) 47 doentes tratados com ECS e 2) 20 doentes tratados com ECRM (todos N+). Os casos do grupo 1 eram clinicamente N0 e, 11 tornaram-se pN+, após o exame histopatológico. Assim, no grupo 1 obtivemos doentes pN+ tratados com ECS e seguimos estes grupos por, no mínimo, 2 anos. O grupo 2 serviu como controle da evolução dos doentes pN+.

**RESULTADOS:**

Nossos resultados mostraram que na comparação do ECS com o ECRM, não houve diferença significativa quanto à evolução (sobrevivência ou recidiva), no entanto, foi possível evidenciar uma pior evolução nos doentes N+ quando comparados aos N0, demonstrando que o principal fator prognóstico é o comprometimento linfonodal. Além disso, apesar do pequeno número de doentes, observamos que os doentes mais idosos e com CEC de faringe tiveram pior evolução e, por isto, talvez não sejam candidatos ao ECS.

**CONCLUSÕES:**

Concluímos, portanto, que o ECS pode ser uma boa opção de tratamento no pescoço N+, em casos selecionados, com metástases cervicais limitadas.

## INTRODUCTION

Lymph node metastasis is one of the most significant prognostic factors for patients with head and neck squamous cell carcinoma (SCC).^1-6^ This topic has been thoroughly studied. Today, several treatment options giving patients a better quality of life without harming patient survival rates have been proposed.

Crile^7^ was the first to systematically describe radical neck dissection, understanding the importance of appropriate treatment for neck lymph nodes in head and neck cancers. This technique was defended and reiterated by Hayes Martin.^8,9^ Currently, radical neck dissection is practically restricted to patients presenting fixed metastases with capsule rupture or in whom exposure of the non-lymphatic structures is evident, along with cases with local tumor recurrence.

At the time when this treatment was described, it was acclaimed in the scientific-medical field. The technique remained unchanged until the 1960s and 1970s, when Suarez and Bocca, respectively, proposed modified radical neck dissection (MRND), which reduced the functional losses but maintained the same oncological result.^10^

Although Crile mentioned the use of dissections of smaller scale than his radical technique,7 albeit with specifications differing from those that are known today, the concept of selective neck dissection (SND) only became popular in the 1980s.10 Classically, SND has been indicated for patients with SCC of the upper aerodigestive tract without clinically detectable metastases (node-negative, N0), but presenting a high risk of developing them.

Some studies have shown that the presence of neck metastasis alone worsens patient prognosis, but that the use of more limited neck dissections, in selected cases, does not seem to harm the evolution and survival rates.^11,12^ Thus, SND, which was first described by Byers, and has already been studied as a therapeutic possibility for node-positive (N+) neck cases in some services, and overall use in cases of N1 necks (neck with one positive linfonode up to 3 cm in diameter) without capsule rupture.^12,13^ Nonetheless, this type of treatment remains controversial, and this has been making some authors believe that randomized studies should be developed to clarify the doubts.

## OBJECTIVE

In this study, our aim was to contribute towards elucidating the doubts regarding the use of more limited neck dissections for patients diagnosed as N+. Through this, the morbidity associated with treatments that are more radical might be avoided. We attempted to evaluate whether SND would be enough for treating patients with SCC of the upper aerodigestive tract who were staged N+, and especially N1. Furthermore, through this, the criteria for using SND and defining groups that are at higher risk of neck recurrence, for which treatments that are more radical should be indicated, might be established.

## METHODS

This was a retrospective study that included patients with SCC in the upper aerodigestive tract (oral cavity, pharynx and larynx) who were surgically treated using SND or MRND (control group) at the Division of Head and Neck Surgery, Department of Surgery, Faculdade de Ciências Médicas da Santa Casa de São Paulo, between 1990 and 2001.

We obtained data by means of a specific protocol, with the aim of investigating the primary tumor site, degree of tumor differentiation, presence of perineural, lymphatic or vascular invasion, presence of neck metastasis, size and number of affected lymph nodes and presence of capsule rupture. From the findings obtained, we stratified the patients into two groups:

- **Group 1:** patients who had undergone SND (n = 47), subdivided into:
  - **1A:** patients with anatomical-pathological test results demonstrating histopatological positive lymph node metastasis (pN+) (n = 11).
  - **1B:** patients without histopatological lymph node metastasis (pN0) (n = 36). In this group, the exclusion criterion was the use of radical or modified radical neck dissection in one side of the neck, with clinically diagnosed metastasis.
- **Group 2 (Control group):** Patients with pN1 (neck with only one metastatic lymph node until 3 cm) or pN2A (neck with one metastatic lymph node from 3 to 6 cm) who underwent MRND (n = 20). In this group, the exclusion criteria was just the presence of neck staging higher than N2A.

We thus observed the clinical evolution of the two groups in relation to neck recurrence and recurrence in the primary site and we analyzed the presence of angiolymphatic invasion, neural invasion and capsule rupture with regard to any associations with neck recurrence and patient evolution. The patient follow-up lasted for at least two years.

The statistical methods used for analyzing the data were: analysis of variance (parametric and non-parametric); randomized Monte Carlo simulations for the chi-squared test; discriminating analysis; correlation analysis; and cluster analysis for variables and patients using the Burnaby Coefficient.

The data on the 47 patients who underwent SND (group 1) and the 20 patients who underwent MRND (group 2) were tested statistically to ascertain their comparability. It was confirmed that the two groups were comparable, i.e. each of them was homogenous from the variance point of view (Levene statistic) and they differed from each other (Brown-Forsythe test).

Patients over 60 years old predominated in group 1. The control group demonstrated similar distribution, which was especially concentrated in the age band over 51 years old. Regarding gender distribution, group 1 had a higher percentage of women (12.76%) than group 2 (5.00%).

We divided the patients into three primary sites: oral cavity, larynx and pharynx. There was greater incidence of patients with SCC in the pharynx in group 2 (30.00% versus 4.25% in group 1).

Thirty patients underwent unilateral SND and seventeen underwent bilateral SND in group 1. In group 2 (control), bilateral neck dissection was performed on nine patients. In all cases, the second side was also an MRND or SND procedure.

It is very important to emphasize that no statistically significant difference was found among the three subgroups, in relation to the duration of follow-up. However, the follow-up for group 1B was slightly longer than for the other two subgroups: in group IB, 66.5% of the patients had a follow-up of 19 to 24 months, while only 36.4% of group 1A had this length of follow-up and 63.3% of group 2.

The loss to follow-up was 9.0% in group 1A, 30.5% in group 1B and 15.0% in group 2.

## RESULTS

Age was not a factor statistically correlated with the appearance of neck recurrence (p = 0.07). However, even though age was not statistically significant, older patients seemed to present a greater chance of developing neck recurrence and, therefore, worse evolution (Table 1).

**Table 1:** Likelihood of neck recurrence among patients with squamous cell carcinoma (SCC) of the upper aerodigestive tract who underwent selective neck dissection (SND) (group 1) and modified radical neck dissection (MRND) (group 2 or control) at the Division of Head and Neck Surgery, Santa Casa de São Paulo, between 1990 and 2001

Age range	Likelihood of neck recurrence
< 40 years	0%
41 - 50 years	7.7%
51 - 60 years	10.5%
> 61 years	24.1%

By stratifying the patients into four age ranges, as shown in Table 1, a tendency for neck recurrence to appear in older age ranges can be seen. Comparing the oldest group with the preceding three groups together, and using data amplification methods, statistical significance of p = 0.002 could be seen with regard to metastasis development in this age range.

The presence of neck recurrence among the three primary sites studied was different (Table 2). It was shown statistically that patients with pharyngeal carcinoma had a greater chance of presenting neck recurrence (p = 0.05). To examine this result more carefully, randomized data amplification methods were used. These showed a correlation between the primary site and the presence of neck recurrence, and this was more common in patients with pharyngeal SCC (p = 0.0001).

**Table 2:** Relationship between primary site of the tumor and the presence of neck recurrence among patients with squamous cell carcinoma (SCC) of the upper aerodigestive tract who underwent selective neck dissection (SND) (group 1) and MRND (group 2 or control) at the Division of Head and Neck Surgery, Santa Casa de São Paulo, between 1990 and 2001

		Without neck recurrence	With neck recurrence	Total
Primary Site	Oral Cavity	27 (p = 0.55)	5 (p = 0.62)	32
	Larynx	21 (p = 0.30)	2 (p = 0.72)	23
	Pharynx	5 (p = 0.64)	3 (p = 0.05)	8
Total		53	10	63

*Note: Among the four patients who are not mentioned, it was impossible to get any information about the presence or absence of neck recurrence.*

Capsule rupture was observed in only one case that underwent SND and in another that underwent MRND. Because of this small sample, no statistically significant relationship could be established between this factor and neck recurrence or patient evolution (p > 0.10).

There was no statistically significant difference between the degree of tumor differentiation and the appearance of neck recurrence or patient evolution. Furthermore, there was no statistical difference between patients with clinical staging of I/II and III/IV in relation to neck recurrence.

**Figure 1:**
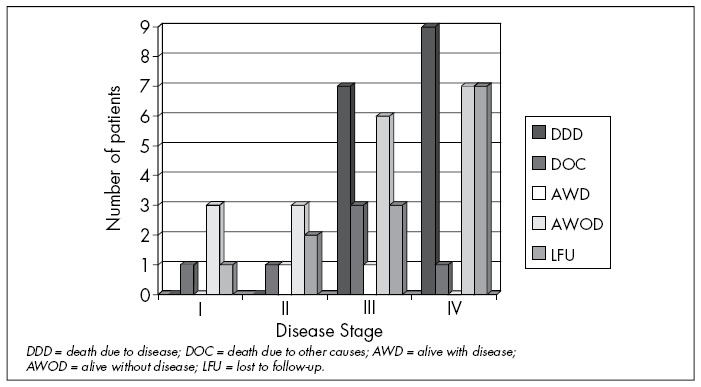
Evolution of patients with squamous cell carcinoma (SCC) of the upper aerodigestive tract who underwent selective neck dissection (SND) or modified radical neck dissection (MRND) at the Division of Head and Neck Surgery, Santa Casa de São Paulo, between 1990 and 2001, according to disease staging.

Among the sample analyzed, the anatomical-pathological staging correlated with patient evolution (p = 0.03), such that there was worse evolution in the higher pathological stages. All the patients who died because of the disease presented pathological staging III or IV, as can be seen in Figure 1.

**Figure 2:**
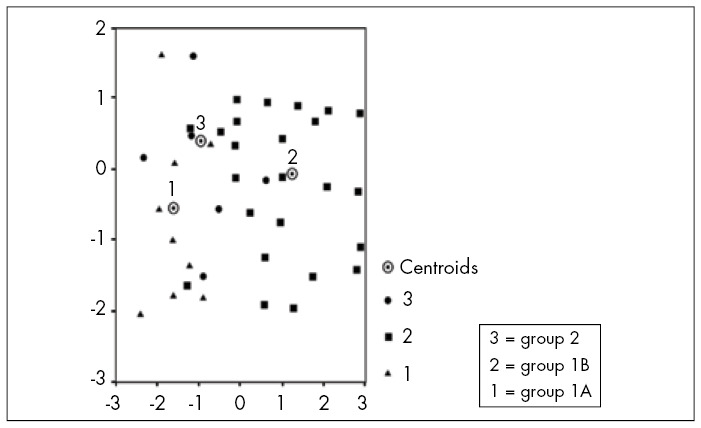
Patients with squamous cell carcinoma (SCC) of the upper aerodigestive tract who underwent selective neck dissection (SND) (group 1) or modified radical neck dissection (MRND) (group 2 or control) at the Division of Head and Neck Surgery, Santa Casa Medical School, São Paulo, between 1990 and 2001, with sequential ordering according to the discriminating variables.

We observed that the presence of positive lymph nodes during the first surgical procedure (p = 0.01) was a factor indicating worse evolution. This became evident by placing the patients from the two groups in sequential order according to the discriminating variables in this study. We found that the patients in groups 1A and 2 (data plotted in triangle and circle, respectively) were spatially much closer than were the patients in group 1B (in square). This shows that patients with node-positive necks behaved in a similar way, independent of the treatment used (Figure 2).

Likewise, Table 3 demonstrates that there was no statistical difference between groups 1A and 2 in relation to patient evolution (N+ patients who underwent SND and N+ patients who underwent MRND, respectively) (p > 0.05). However, between these and group 1B (N0 patients who underwent SND), a statistically significant difference was observed (p < 0.01).

**Table 3:** Statistical analysis on the evolution of patients with squamous cell carcinoma (SCC) of the upper aerodigestive tract who underwent SND (group 1) or modified radical neck dissection (MRND) (group 2 or control) at the Division of Head and Neck Surgery, Santa Casa de São Paulo, between 1990 and 2001

	Tests used	Variables (groups)	Significance
**Evolution**	Tukey HSD	1A -- 1B	p < 0.01
		1A -- 2	p = 0.603
	Scheffé	1A -- 1B	p < 0.01
		1A -- 2	p = 0.631
	Bonferroni	1A -- 1B	p < 0.01
		1A -- 2	p = 1.00

*HSD = honestly significantly different.*

**Figure 3:**
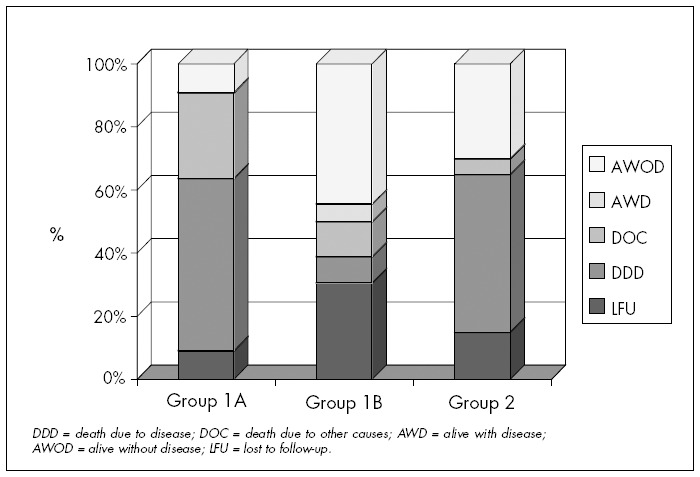
Patients with squamous cell carcinoma (SCC) of the upper aerodigestive tract who underwent selective neck dissection (SND) or modified radical neck dissection (MRND) at the Division of Head and Neck Surgery, Santa Casa de São Paulo, between 1990 and 2001, in percentages.

Figure 3 shows the patient distribution according to the evolution in each of the three groups. Once again, it demonstrates the importance of the neck "status" and not the particular surgical procedure accomplished. In the same way that we did not find any statistically significant difference when analyzing groups 1A and 2 concerning evolution, when patients who died from neck recurrence were analyzed separately (two patients in group 1A and four patients in group 2), no statistically significant difference was observed.

There was no statistically significant difference between the patients who underwent radiotherapy and those who did not, in relation to neck recurrence or evolution.

In our sample, the presence of neck recurrence was a factor related to a poor evolution of the disease. A correlation between neck recurrence and death was present in almost 100% of the cases (p < 0.01). One of the six patients who died from the disease in group 1A did not present any local or neck recurrence, but instead died from cerebral metastasis.

Distant metastases were observed in two patients in group 1, and both were cerebral metastases. Four patients in group 2 presented distant metastases: two cases of pulmonary metastases, one case of pulmonary and osseous metastasis and one case of hepatic metastases.

## DISCUSSION

Diagnosis and adequate treatment for neck metastases are of fundamental importance for patient evolution^14^ and have the aim of avoid possible disease recurrence. On the other hand, today, procedures that are more radical are avoided because of their greater peri and postoperative morbidity and mortality rates, as long as this does not cause a negative impact on disease control.^15^

Several studies have been showing that SND for treating N0 necks and MRND for N+ necks are sufficient for the majority of cases. Careful selection of the type of dissection to be performed, together with judicious indication of postoperative radiotherapy, can improve cure rates as well as the functional and esthetic results.^15^

Some studies have recently suggested and others have demonstrated that SND could be used for selected patients with a node-positive neck when they present clinical staging lower than N2A and when there is no capsule rupture. Some authors have suggested using postoperative radiotherapy.^5,9-24^ According to Andersen et al., SND represents the next logical step towards modified radical neck dissections.^15^

In the Division of Head and Neck Surgery at Santa Casa de São Paulo, SND is classically indicated for treating patients with SCC of the aerodigestive tract without neck metastasis on clinical examination. The patients studied here were, therefore, patients with clinically negative necks, among whom the postoperative histopathological analysis occasionally showed the presence of lymph node metastasis, thus giving rise to restaging as either N1 or N2A. This is, without doubt, a question to be discussed because we believe that a considerable difference exists between patient with clinically negative necks and patient with initial positive staging. However, agreeing with Andersen et al.,^15^ we believe that the same criteria were generally used and there was an absence of capsule rupture and factors that would alter the normal lymphatic flow in the neck (such as previous surgery or radiotherapy) in both patient groups. Therefore, the results found for the patients with clinically negative necks, but microscopically positive findings would encourage us to carry out the procedure for that group.

In our study, we chose patients with primary sites in the oral cavity, larynx and pharynx. This gave rise to "non-uniform methodology" that may have affected the results obtained. However, even though the three sites presented different biological behavior, which would cause different likelihoods of neck metastasis, these cases underwent the same treatment, i.e. MRND, and had the same limited prognosis.

The pharynx was the primary site most frequently related to the development of neck recurrences after the initial tumor treatment (surgery or surgery and radiotherapy). This may suggest that patients with primary tumors located at this anatomical site should sometimes undergo more radical treatments, and may not be candidates for SND even if the neck metastasis presented is only small.

We believe that age is an interesting point to be discussed in this study. Although age was not found to have statistical significance, older patients seemed to present greater chances of developing neck recurrence. Furthermore, patients over 61 years old seemed to have greater chances of this occurring, and this was probably not only attributable to the fact that patients within this age range are more likely to be affected by carcinomas of the upper aerodigestive tract.

Contrary to our findings, Gavilán et al.^3^ found when studying radiotherapy following MRND that age less than 55 years was a negative prognostic factor. The explanation they suggested for these differences was the particular aggressiveness of the tumors and the patients’ immune status.

Several authors have cited the presence of capsule rupture as an indicator of negative prognosis for cases of SCC of the upper aerodigestive tract. This factor is in itself a contraindication for SND in cases of N+ necks, even with the presence of limited metastasis.^2,3,9-13,15,22,25-29^

Because of the limited number of patients in this study, we were not able to correlate the presence of capsule rupture with a greater chance of neck recurrence or negative patient evolution. Nonetheless, from the data presented in the literature and from our data, the use of SND does not seem to be prudent in cases with signs of intraoperative capsule rupture or in the event that frozen-section biopsy confirms the case.

The patients’ staging was analyzed and grouped as I/II and III/IV. No relationship was found between these factors and the development of neck recurrence, although, as is to be expected, staging is related to patient evolution. All of the patients who died from the disease were in stages III or IV. This finding is clearly related to findings of neck metastasis during the initial patient evaluation, which will be discussed later on, since stages III and IV usually include patients with local metastases. Hence, we emphasize the importance of specific persistent follow-up for these patients.

The presence of lymph node metastasis represents the worst prognostic factor for tumors of the head and neck.^3,5,30-32^ Through our study, we saw that findings of affected lymph nodes at the patients’ initial examinations were one of the worst prognostic factors. This is clearly related to negative patient evolution. This worse evolution became evident when the patients were placed in sequence using the discriminating variables selected for our study, which gave a graphic real expression of what occurred in the three groups. Thus, the linking factor between groups was the presence of lymph node metastasis and not the treatment option used. In fact, when we subjected the data to a sophisticated statistical analysis, we did not find any statistical difference between patients who underwent SND with a node-positive neck (group 1A) and those with similar staging (N1 and N2A) who underwent MRND (group 2).

As commented earlier, several authors have been favorable towards SND for neck treatment in selected patients with carcinoma in the different primary carcinoma sites with small and non-fixed neck metastases, and some of them have advocated associating postoperative radiotherapy to the treatment.^5,12,15,17,18,24,33,34^

These authors believed that the rationale would be SND (followed by radiotherapy) for selected patients with limited metastases (N1) located at the first drainage site, but not for each and every type of neck metastasis. Although we have not had the opportunity to evaluate the level of each metastasis in the cases studied in our service, it would seem prudent to observe this simple rule for the indication of SND.

In 2002, Kowalski and Carvalho^11^ conducted a specific study on patients with SCC in the oral cavity with N1 and N2A staging who underwent SDN or MRND. They concluded from analyzing the patterns of disease dissemination through different lymph node levels that SND could be safely performed in patients classified as N1, but that it needed to be used with caution for patients staged clinically as N2A or higher. Furthermore, lymph nodes bigger than three centimeters had a greater chance of presenting capsule rupture. These same authors stated that patients staged as pN+ (positive in the histopathological exam, without palpable lymph nodes) presented a higher rate of neck recurrence, but that this occured independently of the type of neck dissection offered and was possibly related to tumor type and the clinical and pathological staging of the tumor.^11^

Ferlito and Rinaldo^35^ discussed the type of neck dissection to be used in patients with cancer of the larynx and concluded that SND was just as effective as radical or modified radical neck dissection for treating clinically negative necks or those with occult neck metastasis.^35^ The type of neck dissection to be chosen depends, therefore, on the primary tumor and type of local dissemination.

In another paper, Ferlito et al.^28^ noted the importance of elective neck dissection for larynx cancer in relation to prognosis and therapeutic decisions. They once again emphasized that more extensive neck dissections are unnecessary for this type of cancer. They concluded that SND could be used successfully in selected cases with a node-positive neck, particularly in N1 cases and occasionally in N2 cases.^28^

The statistical analysis of our data also demonstrated that there was no difference between patients treated with SND and those treated with MRND in relation to death resulting from neck recurrence. This again confirms our hypothesis that the treatment given to the patients does not influence their evolution. Instead, the determining factor is the presence of neck metastasis at the time of the initial patient evaluation.

Another topic that remains controversial is the indication of postoperative radiotherapy. Several studies have demonstrated the advantages of the use of combined radiotherapy for treating N+ patients who have undergone SND, especially if there are lymph nodes bigger than 3 cm or if there has been capsule rupture.^2,4,12,13,17,20,27,36-38^

In our service, the indication for postoperative radiotherapy for neck treatment does not depend on the type of neck dissection used. It is indicated in cases in which one or more lymph nodes are affected by the disease (> N1) and in the presence of skip metastasis. For the primary tumor, we indicate radiotherapy if the histological analysis demonstrated some degree of malignancy, narrow margins or angiolymphatic and neural invasion.

We did not find any statistical difference between patients who were and were not treated with postoperative radiotherapy, in relation to neck recurrence or disease evolution. This may once again be due to the small sample that we were studying and we believe that this result should be disregarded. Randomized studies must be conducted to establish which approach is more appropriate.

In our opinion, patients who present one or more positive lymph nodes or who demonstrate anomalous patterns of lymph node dissemination, such as neck metastasis outside the first drainage site and capsule rupture, should be treated with complementary radiotherapy. We believe that these patients, as suggested by some other studies, would still have a worse evolution than would those patients who did not undergo radiotherapy. This is not obviously related to the radiotherapy itself, but to other factors that lead to its indication. In our study, we were not able to evaluate these findings in our sample.

Our study was able to determine that the appearance of neck recurrence over the course of the patients’ evolution is a factor indicative of negative evolution, and is almost 100% correlated with death. Other authors have also reported that the appearance of neck recurrence correlated with low success rates in neck recovery, as well as emphasizing that the main factors relating to recurrence are capsule rupture and the presence of multiple lymph nodes at several levels.^20^ We should again emphasize the importance of optimizing the proposed treatment at the time of the first operation, but avoiding "over treatment", which can cause sequelae that could worsen the patients’ quality of life.

We did not find any statistically significant differences in relation to the length of patient follow-up. Group 1B, with a greater number of patients and better prognosis presented the highest rate of discontinuation of follow-up among our entire patient population (30.5%). In group 1A, only one patient (9.0%) did not continue with the follow-up in our service, along with three patients (15.0%) in group 2.

Therefore, our study allows us to believe that the following are factors favoring worse evolution for patients with malignant neoplasia of the head and neck:

- Age over 61 years;
- Primary tumor in the pharynx;
- Neck metastasis at the time of the initial patient evaluation;
- Neck recurrence.

Consequently, perhaps it would be more suitable to indicate treatment that is more radical for older patients with a primary tumor in the pharynx. However, we do not have sufficient data to reach this conclusion. When SND was performed on patients with limited metastases (< N2A), it did not have any influence on the development of neck recurrence or on patient evolution.

## CONCLUSION

According to our findings from this study, we can conclude that SND may be suitable for treating N+ necks, particularly when staged as N1 without capsule rupture. Older patients with primary pharyngeal tumors seem to present worse evolution, thereby suggesting that SND would not be the most appropriate treatment option for these patients. The groups at higher risk of developing neck recurrence are: age over 61 years, primary tumor located in the pharynx and neck metastasis diagnosed during the patients’ first treatment.
